# Functionalization of eggshell membranes with CuO–ZnO based p–n junctions for visible light induced antibacterial activity against *Escherichia coli*

**DOI:** 10.1038/s41598-020-78005-x

**Published:** 2020-12-01

**Authors:** Nicoleta Preda, Andreea Costas, Mihaela Beregoi, Nicoleta Apostol, Andrei Kuncser, Carmen Curutiu, Florin Iordache, Ionut Enculescu

**Affiliations:** 1 National Institute of Materials Physics, Atomistilor 405A, 077125 Magurele, Romania; 2 Microbiology Immunology Department, Faculty of Biology, University of Bucharest, Aleea Portocalelor 1-3, 060101 Bucharest, Romania; 3 University of Agronomic Sciences and Veterinary Medicine of Bucharest, 011464 Bucharest, Romania

**Keywords:** Surfaces, interfaces and thin films, Organic-inorganic nanostructures

## Abstract

Biopolymers provide versatile platforms for designing naturally-derived wound care dressings through eco-friendly pathways. Eggshell membrane (ESM), a widely available, biocompatible biopolymer based structure features a unique 3D porous interwoven fibrous protein network. The ESM was functionalized with inorganic compounds (Ag, ZnO, CuO used either separately or combined) using a straightforward deposition technique namely radio frequency magnetron sputtering. The functionalized ESMs were characterized from morphological, structural, compositional, surface chemistry, optical, cytotoxicity and antibacterial point of view. It was emphasized that functionalization with a combination of metal oxides and exposure to visible light results in a highly efficient antibacterial activity against *Escherichia coli* when compared to the activity of individual metal oxide components. It is assumed that this is possible due to the fact that an axial p–n junction is created by joining the two metal oxides. This structure separates into components the charge carrier pairs promoted by visible light irradiation that further can influence the generation of reactive oxygen species which ultimately are responsible for the bactericide effect. This study proves that, by employing inexpensive and environmentally friendly materials (ESM and metal oxides) and fabrication techniques (radio frequency magnetron sputtering), affordable antibacterial materials can be developed for potential applications in chronic wound healing device area.

## Introduction

The development of affordable wound care dressings based on biopolymers by employing cost-effective and environmentally safe materials and techniques of fabrication gained an extensive attention from the scientific community^1–5^. Widely available, inexpensive, biocompatible, biodegradable, non-toxic and renewable, the biopolymers are structural components of the tissues of living organisms and in some cases exhibit biochemical properties that facilitate the wound healing processes^1,2,5–7^.

A natural polymer and an abundant industrial and domestic waste, the eggshell membrane (ESM) is currently regarded as an interesting platform for designing functionalized architectures with potential applications in water purification^8^, actuators^9^, photocatalysis^10^, wound healing products area^11–20^, etc., some progress being made for its industrial valorization^21–23^. ESM presents a unique interlaced fibrous 3D network structure formed by fibers with 80–85% proteins in their composition, of which 10% are collagens (types I, V and X) and 70–75% are other proteins and glycoproteins (hyaluronic acid, glucosamine)^24^. Additionally, ESM possesses a set of essential intrinsic characteristics such as flexibility, high water retention capacity, large surface area and high porosity which favors the diffusion of gas and water molecules and enable the attachment and proliferation of the cells. Due to these features, ESM is capable of mimicking the physico-chemical and biological characteristics of the extracellular matrix and performs similar key functions during wound healing of skin injuries. Hence, ESM can provide a biological stimulus in this process, being a low-cost alternative to currently wound care products employed for mitigating bacterial infection and for accelerating the wound healing. Furthermore, the functionalization of ESM with inorganic compounds (metals, metal oxides or metal-decorated metal oxides) with already demonstrated antibacterial effect provides a solution to the need for a wound dressing with enhanced functionality. An ideal wound dressing should have simultaneously a good biocompatibility and an adequate antibacterial activity, protecting the injured area from infection and providing a favorable environment (moisture, oxygen and water vapor permeability) for tissue regeneration. However, as far as we know, only three research papers report on the decoration of ESM with inorganic structures with already known antibacterial properties: Ag ^18,19^ and Ag-ZnO ^17^. It is acknowledged that silver prevents bacterial infections in wounds^25,26^, but for both cost and efficiency related reasons, nanocomposites based on Ag and metal oxides like Ag-ZnO ^27^, Ag-CuO ^27^, Ag and CuO impregnated on Fe doped ZnO ^28^, Cu_2_O and Ag co-modified ZnO ^29^, etc. have been recently synthesized by various wet chemical methods. Some metal oxides such as ZnO and CuO exhibit also a pronounced antibacterial activity either as single components^30^ or as nanocomposites^31–33^. The two metal oxides present a high potential for application in the field of antibacterial products considering characteristics which include low-price, minimal environmental impact, morphologically rich families of nanostructures which can be relatively easily synthesized by various approaches^34–37^. Moreover, ZnO is a n-type metal oxide characterized by a wide band gap (~ 3.37 eV) ^36^, while CuO is a p-type metal oxide with a narrow band gap (generally in the range of 1.2 to 2.16 eV) ^37^. By joining them, a p–n junction can be obtained, a structure which, separates into components the charge carrier pairs produced by visible light irradiation. Further, this process can enhance the generation of highly reactive oxygen species (ROS), these being key mediators of the material’s bactericide effect. Additionally, the formation of a p–n junction between ZnO and other semiconductors is an interesting strategy to improve the ZnO stability and its photocatalytic activity, being known that the environmental factors such as solution pH, dissolved oxygen level, particle morphology and UV irradiation can have a significant effect on the ZnO stability during catalysis process^38^. Accordingly, recent papers focused on the visible-light photocatalytic activity of various p-n heterojunctions based on ZnO such as ZnO/CuO ^39,40^, Bi_2_S_3_/ZnO ^41^, ZnO/ZnBi_2_O_4_^42^ or ZnO/CuBi_2_O_4_^43^ shown that such compounds are featured by a good stability maintaining their photocatalytic ability. Moreover, some reports regarding the photocatalytic activity of ZnO deposited by radio frequency (RF) magnetron sputtering put in evidence that the obtained ZnO nanostructured layers are stable even under UV irradiation and can be used as reusable photo-catalysts in aqueous reactions under a wide range of pH medium for photocatalytic applications^44,45^.

Lately, various physical deposition methods and chemical synthesis were used for designing and developing new architectures based on organic and inorganic compounds for applications in solar cells, photocatalysis, antimicrobial agent domain, optoelectronic devices, biomedical field, etc. ^46–48^. A versatile, high-yield and low-temperature physical vapor deposition technique, magnetron sputtering was employed for preparing thin films on a wide range of substrates. The films are usually polycrystalline, uniform and present good adhesion to the surface of the substrates. Therefore, sputter coating with metal or metal oxide layers can add or enhance antibacterial properties to the biopolymer fibers’ surface^49^ or to the non-woven fabrics’ surface^50^. Consequently, by sputter coating one can improve the functionality of wound dressing materials. In contrast to the decoration of ESM with inorganic nanostructures through wet chemical methods, the coating and/or decoration by RF magnetron sputtering can results in an inorganic layer with both improved quality and function.

In this context, the present study is focused on the functionalization of eggshell membranes (ESMs) with inorganic materials (Ag, ZnO, CuO used either separately or combined) by using RF magnetron sputtering, a straightforward and scalable deposition technique. In a first step, the ESMs were coated with either Ag, ZnO or CuO layer. Further, the metal oxide-coated ESMs were decorated with Ag nanoparticles for achieving a synergistic antibacterial effect. ESMs covered with metal oxides layers, namely ZnO-coated ESM and CuO-coated ESM were decorated with CuO and ZnO nanoparticles, respectively, in order to form a type of junction which was expected to enhance the antibacterial activity. The morphological, structural, compositional, surface chemistry, optical, cytotoxicity and antibacterial properties of all functionalized ESMs were evaluated and compared to each other. It was found that for the CuO–ZnO based axial p-n junctions, visible light irradiation leads to an enhanced antibacterial activity when tested on *Escherichia coli* (*E. coli*). This antibacterial activity is superior to that observed in the case of ESMs functionalized with silver or with a single type of metal oxide. This is most probably a consequence of charge carrier pairs generated by irradiation and separated in the internal field leading to the development on the surface of ROS which are responsible for bactericide effect.

## Experimental section

### Preparation of functionalized eggshell membranes

The eggshell membranes (ESMs) were obtained from commercial fresh hen eggs bought from local supermarkets. In the functionalization of ESMs by RF magnetron sputtering, silver (99.99% purity), zinc oxide (99.9% purity) and copper oxide (99.7% purity) sputtering targets with 2 inch in diameter and 0.125 inch in thickness were used, being purchased from Kurt J. Lesker Company Ltd. (UK).

In the first step, ESMs were obtained by manually stripping them from the broken eggshells and washing several times with distilled water. Then, the wet ESMs were mechanically fixed between two overlapping stainless steel frames in order to prevent twisting during the drying process in air at ambient conditions. In the second step, the dried ESMs with dimensions of 2 cm × 3 cm were taken off the supports and functionalized only on one side with metal oxide or/and metal by RF magnetron sputtering (Tectra GmbH Physikalische Instrumente). Hence, for assuring a uniform deposition of the inorganic compounds on the surface of the organic substrates, the ESMs samples were disposed in a circle at an equal distance from the centre of the sample holder (16 cm diameter), this being rotated (4 rot/min) during the deposition process. The vertical distance between the target and the sample holder was 12 cm, the target being pre-sputtered for 20 min, before each deposition, in order to remove any possible contamination from its surface. The deposition process was carried out in an Ar atmosphere with a purity of 9.6 (99.9999%) from Linde. The power applied on the magnetron was 30 W (Ag) or 100 W (ZnO, CuO). During the deposition, the pressure in the chamber was 4.6 × 10^−3^ mbar (Ag) or 5.4 × 10^−3^ mbar (ZnO, CuO). For coating the ESMs with a continuous and compact layer, the deposition time was 1 h (Ag) or 3 h (ZnO, CuO). For the decoration with nanoparticles of the layer coated-ESMs, the deposition time was 4 min (Ag) or 6 min (ZnO, CuO).

**Figure 1:**
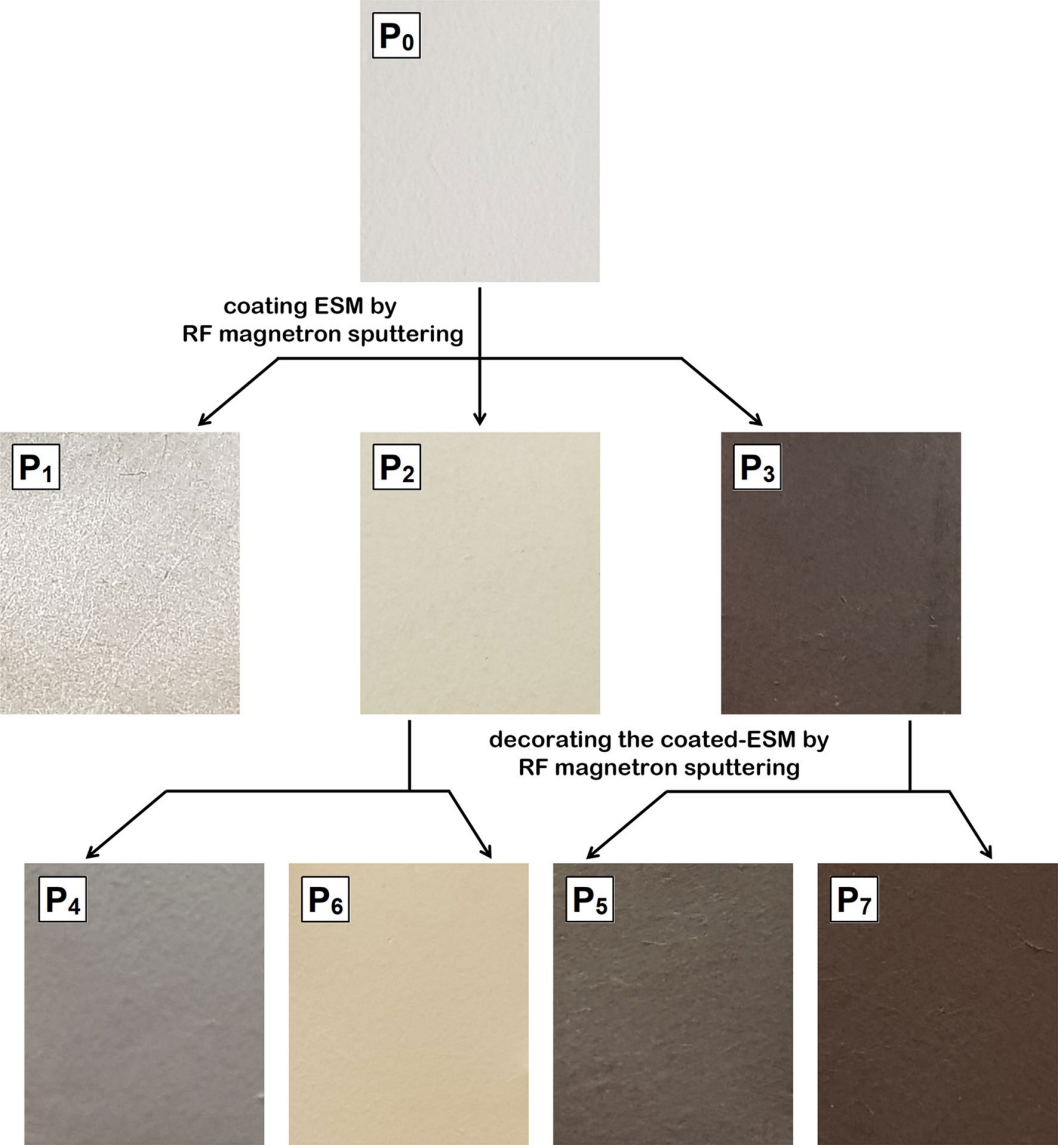
Optical images of native ESM (P_0_) and functionalized ESMs (P_1_–P_7_).

Images of the investigated samples are presented in Fig. 1, being labeled as follows: P_0_ (native ESM), P_1_ (Ag-coated ESM), P_2_ (ZnO-coated ESM), P_3_ (CuO-coated ESM), P_4_ (ZnO-coated ESM decorated with Ag nanoparticles), P_5_ (CuO-coated ESM decorated with Ag nanoparticles), P_6_ (ZnO-coated ESM decorated with CuO nanoparticles) and P_7_ (CuO-coated ESM decorated with ZnO nanoparticles).

The visual/color detail is specific for each deposited layer, the white–ivory color of the native ESM being changed in metallic grey (P_1_), beige (P_2_), brown (P_3_), metallic beige (P_4_), metallic brown (P_5_), sand beige (P_6_) or dark brown (P_7_). Moreover, after functionalization, the surface of ESM is completely and uniformly coated without the appearance of cracks on its surface.

### Characterization techniques

A complex characterization of the functionalized ESMs was completed using a set of complementary techniques. The morphology and the elemental composition were analyzed using a Zeiss Merlin Compact field emission scanning electron microscope (FESEM) and a Zeiss EVO 50XVP scanning electron microscope equipped with an energy dispersive X-ray analysis (EDX) QUANTAX Bruker 200 accessory. At nanoscale level, the morphology, atomic structure and local chemical composition were investigated by high-resolution transmission electron microscopy (HRTEM), EDX including elemental mapping in scanning transmission electron microscopy (STEM) and by selected area electron diffraction (SAED) using a Cs probe-corrected JEM ARM 200F analytical electron microscope.

The crystalline phase was identified by X-ray diffraction (XRD) using a Bruker AXS D8 Advance instrument with Cu Kα radiation (λ = 0.154 nm), the source being operated at 40 kV and 40 mA. The surface chemistry was evaluated by X-Ray photoelectron spectroscopy (XPS) performed in an AXIS Ultra DLD (Kratos Surface Analysis) setup, using Al K_α1_ (1486.74 eV) radiation produced by a monochromatized X-Ray source at operating power of 225 W (15 kV × 15 mA). The base pressure in the analysis chamber was 1.0 × 10^–8^ mbar. Charge compensation was reached by using a flood gun operating at 1.5 A filament current, 2.7 V charge balance, 1.0 V filament bias. High resolution core level spectra have been recorded using hybrid lens mode, 40 eV pass energy and slot aperture. In the deconvolution of the core level spectra Voigt profiles (singlets or doublets) were used, based on previously described methods^51^. The optical properties were investigated by reflectance employing a Perkin-Elmer Lambda 45 UV–VIS spectrophotometer equipped with an integrating sphere. The following software programs were involved in the data acquisition: Zeiss SmartSEM (FESEM), Zeiss SmartSEM and Bruker ESPRIT (SEM/EDX), Olympus Imaging and Image view (HRTEM/STEM/SAED), Bruker DIFFRAC plus BASIC (XRD), Perkin-Elmer UV WINLab (reflectance), Vision (XPS). The analysis of the XPS data was performed using the Igor program, the other experimental data being analyzed by Origin Pro 2017 program.

### Biological assessment

The specific properties of the functionalized ESMs were evaluated from cytotoxicity and antibacterial activity perspective. Thus, cytotoxicity was investigated using fluorescent microscopy, MTT assay and oxidative stress assessment. The tests were carried on human mesenchymal amniotic fluid stem (AFS) cells. These are well suited for biocompatibility tests due to their features such as viability, proliferation, protection against the body immune system and differentiation capacity into different types of tissue.

A qualitative evaluation of the AFS cell viability was acquired by fluorescence microscopy (Thermo Fischer Scientific) using the RED CMTPX fluorophore. In DMEM culture medium and in the presence of the investigated sample, the RED CMTPX dye was added at a final concentration of 5 μM and incubated for 30 min to allow the dye to penetrate the cells. After 5 days, the AFS cells were washed with phosphate buffered saline and visualized, the photomicrographs being taken with an Olympus CKX 41 digital camera driven by CellSense Entry software.

A quantitative evaluation of the AFS cell viability was obtained using a Vybrant MTT Cell Proliferation Assay Kit (Thermo Fischer Scientific). The AFS cells were cultured in DMEM medium supplemented with 10% fetal bovine serum, 1% penicillin and 1% streptomycin antibiotics (all from Sigma-Aldrich). In order to keep the optimal culture conditions, the culture medium was changed twice a week. Hence, the AFS cells were grown, for 72 h, in 96-well plates, with a seeding density of 3000 cells/well in the presence of the investigated samples. Then, 15 ml solution I (2 mM MTT) was added and incubated at 37 °C for 4 h. Next, for dissolving the formed formazan crystals, solution II (1 mg sodium dodecyl sulfate in 10 ml 0.01 M HCl) was added by pipetting it vigorously. After 1 h, the absorbance at 570 nm wavelength was recorded using a TECAN Infinite M200 spectrophotometer.

The oxidative stress for AFS cells was analyzed by GSH-Glo Glutathione Assay Kit (Promega). The AFS cells were seeded for 24 h at a density of 3000 cells in 300 μl of DMEM culture medium supplemented with 10% fetal bovine serum and 1% antibiotics (penicillin, streptomycin/neomycin) in 96-well plates and in the presence of the investigated sample. Afterwards, 100 μl 1X GSH-Glo Reagent was added and incubated at 37 °C for 30 min. Then, 100 μl Luciferin Detection Reagent was also added and incubated at 37 °C for another 15 min. Subsequently, the cells were homogenized and the plate was investigated using a Microplate Luminometer Centro LB 960.

Further, the antibacterial activity was evaluated against non-pathogenic *Escherichia coli* (*E. coli* ATCC 25922) strain, as Gram-negative bacteria model, in both planktonic and biofilm growth state. Thus, for planktonic cells, 2 ml tryptic soy broth (TSB) and 20 μl microbial suspension with 0.5 McFarland density (1.5 × 10^8^ CFU/mL) were added on the sterilized investigated samples placed in 6-well plates. After 24 h incubation at 37 °C, the turbidity of the culture medium was determined by measuring the absorbance at 620 nm with a Biotek Synergy-HTX ELISA multi-mode reader spectrophotometer. In the case of biofilms, after incubation, the investigated samples were washed in sterile saline solution in order to remove the non-adherent bacteria, while the adherent bacteria remained attached to the respective samples. Next, they were introduced into Eppendorf tubes with 1 ml sterile saline and vortexed for 30 s. From the suspension recovered in sterile saline, decimal dilutions were obtained and seeded in triplicate (3 replicates of 10 µl each) on agar medium in order to calculate the number of CFU (colony forming units)/ml. The antibacterial activity of the functionalized ESMs was also evaluated under exposure to the visible light which was provided by a commercially LED lamp, Vorel 82730 (100 mW power). In this case, the sterilized investigated samples were placed in 6-well plates containing ~ 10^5^ CFU/ml microbial suspension, the incubation being carried out at 37 °C for 3 h, 6 h and 9 h under illumination with the LED lamp. At the end of each incubation time, decimal dilutions obtained from the suspension recovered in sterile saline were seeded in triplicate (3 replicates of 10 µl each) on agar medium for evaluating the number of colonies. An *E. coli* solution used as a control sample was investigated in the same experimental conditions but in the absence of a functionalized ESM. According with a previous article^52^, the antibacterial efficacy of the investigated samples was calculated in terms of log reduction (LR) and percentage of bacterial cell reduction (R, %) using the following equations:

$$ {\text{LR }} = {\text{ log}}_{{{1}0}} \left( {{\text{untreated viable cell density}}/{\text{treated viable cell density}}} \right) $$

R % = [(CFU_c_-CFU_p_)/CFU_c_] × 100%; where CFU_c_ and CFU_p_ represent the numbers of CFU/ml for the control and each investigated sample.

It has to be mentioned that the biological tests were carried out in three replicates. Each replicate was performed in triplicate. The experiments were repeated two times in triplicates. In the biological assays, the statistical data were analyzed using the Origin Pro 7.5 software.

## Results and discussions

**Figure 2:**
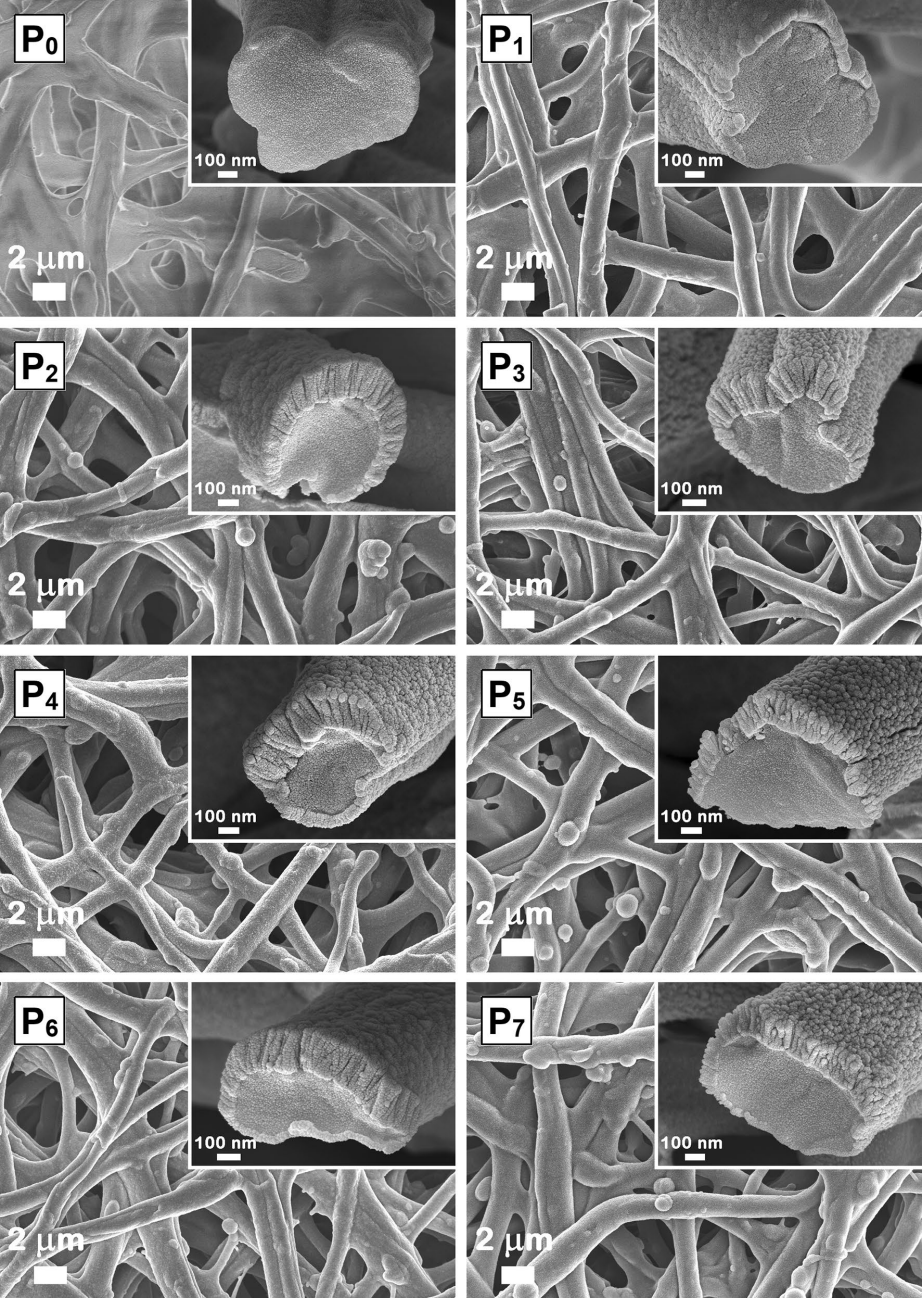
FESEM images of native ESM (P_0_) and functionalized ESMs (P_1_–P_7_). Insets: cross-sectional FESEM images of the corresponding samples.

**Figure 3:**
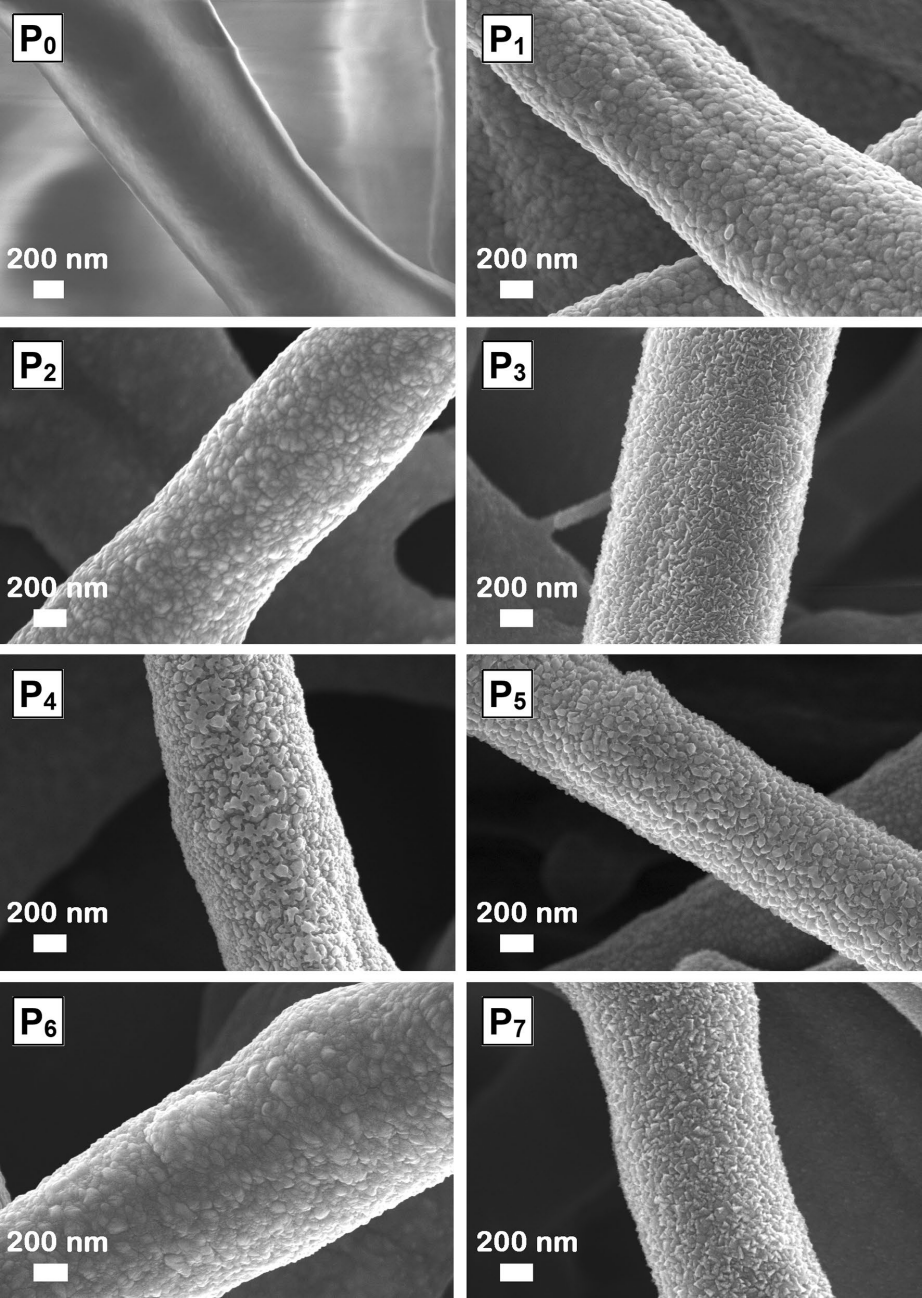
FESEM images at higher magnification of native ESM (P_0_) and functionalized ESMs (P_1_–P_7_).

The morphology of native and functionalized ESMs was analyzed by FESEM, the images being presented at low and high magnification in Figs. 2 and 3, respectively. Thus, the structure of P_0_ sample consists in a macroporous network featuring highly cross-linked smooth fibers with diameter sizes in the micrometer range and pores of several micrometers.

The FESEM images of all functionalized ESMs, P_1_–P_7_ sample (Fig. 2), indicate that the typical 3D porous network of the ESM is successfully preserved during its functionalization, the deposition parameters employed in the RF magnetron deposition process being adequate chosen for avoiding the embedding of the ESM fibers into a metallic or metal oxide thick layer. A continuous and uniform film of either metal or metal oxide was deposited on the surfaces of the fibers.

The FESEM images at higher magnification of all functionalized ESMs (Fig. 3) reveal that P_1_, P_2_ and P_3_ samples are coated with nanostructured self-assembled films consisting of nanoparticles having a relatively homogeneous size, ~ 15 nm (Ag) and ~ 20 nm (ZnO and CuO).

The cross-section FESEM images (Fig. 2 insets) emphasize the hybrid structure based on an organic core and an inorganic shell. Also, from these images, the thickness of the inorganic shell was estimated as being ~ 130 nm for P_1_ sample, ~ 300 nm for P_2_ and P_3_ samples and ~ 320–330 nm for P_4_–P_7_ samples. For improved resolution in the case of the cross-section FESEM imaging, the deposition of a thin layer of gold (~ 20 nm) was necessary.

**Figure 4:**
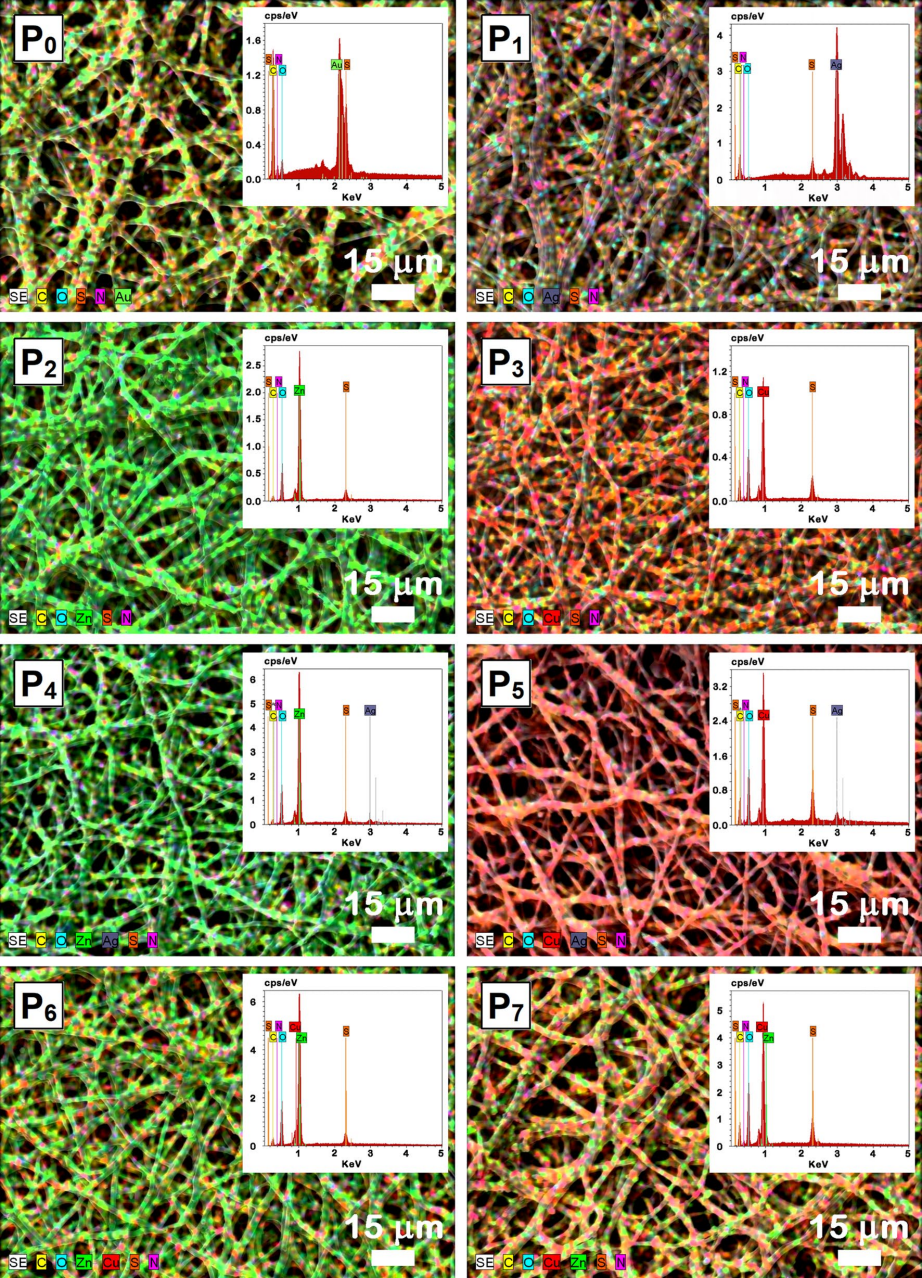
EDX mapping images of native ESM (P_0_) and functionalized ESMs (P_1_–P_7_). Insets: EDX spectra of the corresponding samples.

The elemental distribution on the ESM surface and the composition of the samples were evaluated by EDX spectroscopy, the mapping images and the corresponding spectra being given in Fig. 4. The EDX mapping images illustrate that all functionalized ESMs present a uniform distribution of the chemical elements on their surfaces. The EDX spectrum of P_0_ sample reveals the presence of the typical elements, C, N, O and S, which can be found in the ESM structure^18^. In addition, a signal corresponding to Au is also observed because, as we already mentioned, a thin layer of gold was sputtered on the surface of the native ESM prior to its morphological investigation. In the EDX spectra of P_1_–P_7_ samples, the simultaneously presence of the signals corresponding to the ESM elements with those associated to Ag in P_1_, P_4_ and P_5_ samples, to Zn in P_2_, P_4_, P_6_ and P_7_ samples and to Cu in P_3_, P_5_, P_6_ and P_7_ samples confirms the ESM functionalization. Based on the EDX analysis, the atomic percentage of the elements contained in the functionalized ESMs was evaluated (Table S1). Thus, in the inorganic compact layer, the atomic percentage was estimated at ~ 18% for Ag and between 32 and 38% for Zn and Cu while in the case of the decorated inorganic nanoparticles, the atomic percentage was evaluated at ~ 1% for these three elements.

**Figure 5:**
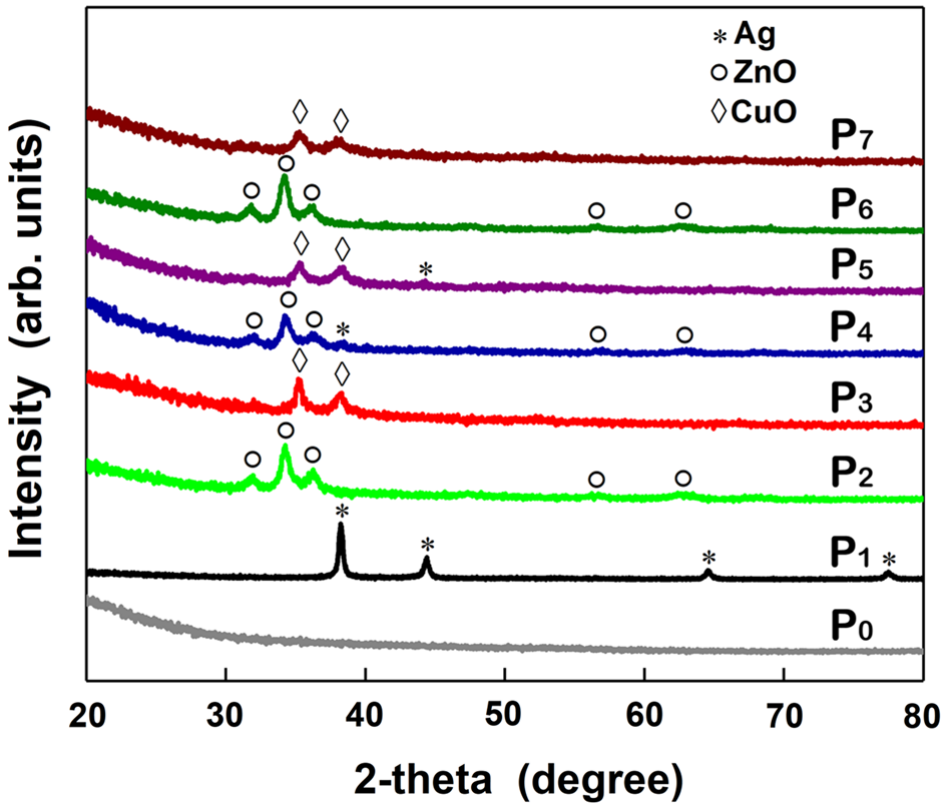
XRD patterns of native ESM (P_0_) and functionalized ESMs (P_1_–P_7_).

The structural information of the native and functionalized ESMs samples was obtained by XRD analysis (Fig. 5). The XRD pattern of P_0_ sample indicates the amorphous character of ESM related to its composition based on amine, amides and carboxylic compounds.

The peaks at 2θ: 38.2°, 44.4°, 64.6° and 77.5° observed in the XRD pattern of P_1_ sample are indexed to (111), (200), (220) and (311) planes of the face-centered cubic Ag structure (JCPDS 04–0783). The XRD patterns of P_2_ sample exhibit peaks at 2θ: 31.9°, 34.2°, 36.3°, 47.3°, 56.7°, 62.6° and 68.0° associated to (100), (002), (101), (102), (110), (103) and (112) planes of the hexagonal wurtzite ZnO structure (ICDD 00-035-1451). The XRD pattern of P_3_ sample displays peaks at 2θ: 35.2° and 38.7^o^ corresponding to (110) and (11–1) planes of the monoclinic CuO structure (ICDD 00-048-1548).

In the XRD patterns of P_4_ and P_5_ samples, the presence of peaks at ~ 38.5° (P_4_) and ~ 44.3° (P_5_) prove the existence of the Ag nanoparticles on the ZnO layer (P_4_) and CuO layer (P_5_), respectively. However, in the XRD patterns of P_6_ and P_7_ samples, the diffraction signature of the metal oxide nanoparticles employed for the decoration had not been detected, only the characteristic diffraction peaks assigned to the metal oxide which form the compact layer deposited on the surface of ESM, hexagonal wurtzite ZnO (P_6_) and monoclinic CuO (P_7_), respectively being clearly identified.

**Figure 6:**
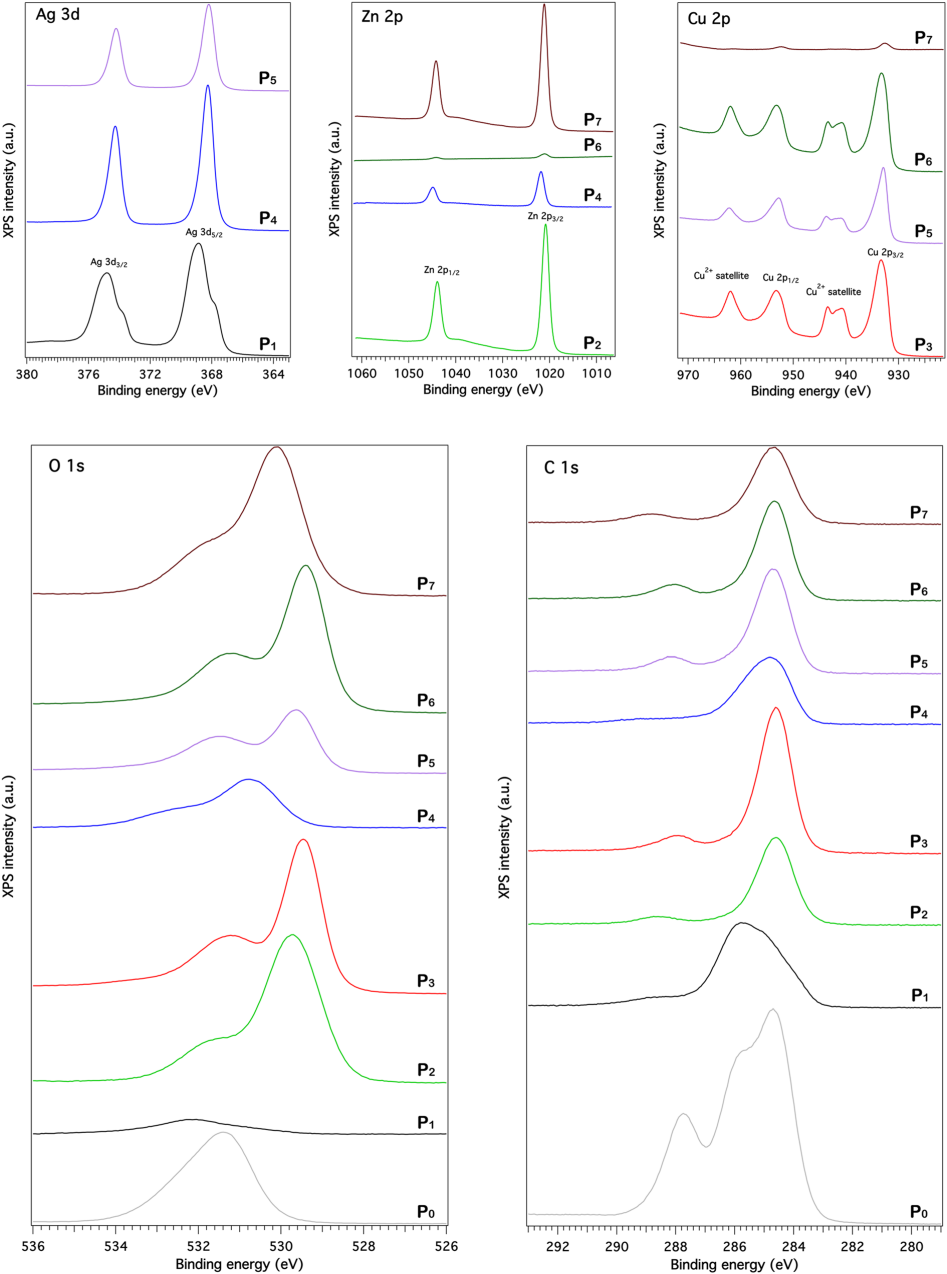
XPS spectra for the Ag 3d, Zn 2p, Cu 2p, O 1s and C 1s core levels in native ESM (P_0_) and functionalized ESMs (P_1_–P_7_). Artificial intensity offsets are introduced for clarity.

Therefore, in order to certify the chemical composition of the inorganic shell and the oxidation states of each constituent, all samples were investigated by XPS, their spectra being presented in Fig. 6. The peaks observed in the XPS spectra of Ag 3d, Zn 2p and Cu 2p were indexed as follows: (i) the peaks at ~ 368 eV and ~ 374 eV to Ag 3d_5/2_ and Ag 3d_3/2_ levels of Ag (0)^53,54^; (ii) the peaks at ~ 1021 eV and ~ 1045 eV to Zn 2p_3/2_ and Zn 2p_1/2_ levels of Zn^2+^ in ZnO^40,55^ and (iii) the peaks at ~ 933 eV and ~ 954 eV to Cu 2p_3/2_ and Cu 2p_1/2_ levels of Cu^2+^ in CuO^40,55^. For native ESM, the broad peak at ~ 531.5 eV from the XPS spectra of O 1 s represents the different oxygen environments in the protein amino acids^56,57^ while the peaks at ~ 284.5 eV, ~ 285.5 eV and ~ 287.5 eV from the XPS spectra of C 1s can be correlated to C–C, C–N and C=O bonds, respectively^56,57^. In the XPS spectra of O 1s for functionalized ESMs, the broad peak at ~ 529–530 eV can be attributed to O^2−^ state in metal oxides (ZnO and CuO)^40,55^. It can be noticed the shift of this peak to ~ 531 eV, responsible for this shift being, most probably, a contamination of the surfaces with carbonate from the environment. Moreover, the deconvolution obtained by Voigt profiles of the core level spectra of Ag 3d (Figure S1), Zn 2p (Figure S2) and Cu 2p (Figure S3) were also analyzed for evidencing the presence of other species of these inorganic elements. Thus, the deconvolution of the XPS spectra for the core level Ag 3d_5/2_ shows a broad peak with two components confirming the hypothesis concerning the existence of more than one silver species. The signal centered at 368.0 eV (P_1_) or 368.2 eV (P_4_ and P_5_) confirms the existence of free metallic silver atoms (Ag (0)), in agreement with reported data for Ag foil^53^ and Ag nanoparticles^54^. Meanwhile, the signal centered at 369.0 eV (P_1_), 368.9 eV (P_4_) or 368.8 eV (P_5_) can indicate positively charged silver atoms (Ag (0) + δ), these resulting, as it is suggested in the literature, from the slight oxidation in air of the metallic atoms situated on the surface of the silver layer and exposed to ambient conditions^58,59^ or from the silver interaction with a relatively high electronegative atom (oxygen, nitrogen or sulphur)^56,60,61^. It is also worth noticing that comparatively with the binding energy indexed to the metallic silver, the oxidized Ag-bulk is characterized by lower binding energy while, in striking contrast, the air-exposed Ag nanoparticles exhibit higher binding energy^62^. The deconvolution of the XPS spectra for the core level Zn 2p_3/2_ reveals a narrow peak centred at 1020.9 eV (P_2_), 1021.9 eV (P_4_), 1021.1 eV (P_6_) or 1021.2 eV (P_7_) assigned to Zn^2+^ state in ZnO, consistent with the previously reported data^40,55^. The deconvolution of the XPS spectra for the core level Cu 2p_3/2_ exhibits a broad peak with two components, one centered at 933.1 eV (P_3_), 932.9 eV (P_5_), 933.0 eV (P_6_) or 932.6 eV (P_7_) associated with Cu^2+^ state in CuO, in accordance with reported data^40,55^ and other at 934.9 eV (P_3_), 934.8 eV (P_5_), 934.8 eV (P_6_) or 934.5 eV (P_7_) linked most probably to a slight contamination of the surfaces with carbonate from the environment. Additionally, the formation of CuO is demonstrated by the presence of the satellite peaks at ~ 940–945 eV and ~ 964 eV characteristic only to the bivalent oxidation state of Cu^40,55^. Consequently, the XRD data and the XPS results confirm the presence of Ag in P_1_, P_4_ and P_5_ samples, ZnO in P_2_, P_4_, P_6_ and P_7_ samples and CuO in P_3_, P_5_, P_6_ and P_7_ samples.

**Figure 7:**
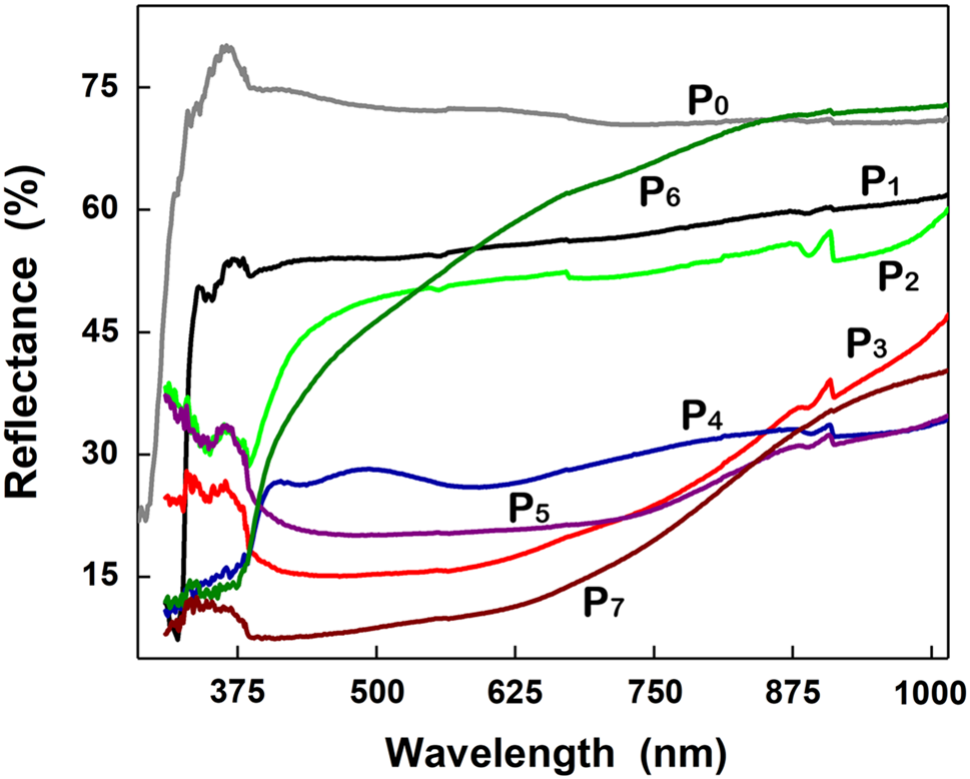
Reflectance spectra of native ESM (P_0_) and functionalized ESMs (P_1_–P_7_).

The optical properties of the native and functionalized ESMs were analyzed by diffuse reflectance (Fig. 7). In the case of P_0_ sample, the reflectance spectrum displays a decrease at ~ 330 nm, in accordance with that reported in the literature for the native ESM^63,64^. A weak decrease at ~ 405 nm is barely noticed in the reflectance spectrum of P_1_ sample owed to a weak surface plasmon resonance (SPR) effect induced by the Ag film deposited on the surface of the ESM^65^. In the reflectance spectra of P_2_ and P_3_ samples, a strong decrease can be observed below ~ 420 nm (P_2_) and ~ 860 nm (P_3_) due to the band-to-band transition in ZnO and CuO, respectively, in agreement with those previously reported^36,37^.

The reflectance spectra of P_4_–P_7_ samples are dominated by the optical signature of the metal oxide layer deposited on the ESM surface, ZnO (P_4_ and P_6_) or CuO (P_5_ and P_7_). However, in the case of P_4_, additionally to the strong decrease peak related to the ZnO, two characteristic plasmon bands are clearly identified at ~ 435 nm and ~ 590 nm, similar with those observed in other studies^66,67^. The result can be explained taking into account that in this case the Ag nanoparticles decorate the surface of the metal oxide-coated ESM in comparison with P_1_ where the Ag nanoparticles formed a compact and uniform film on the surface of the ESM. It is noteworthy that the presence, position and intensity of the SPR band associated to the Ag nanoparticles is strongly influenced by many parameters of the nanoparticles such as size, shape, inter-particle distance, surface chemistry, surrounding environment, etc^68^.

Using the reflectance data and Kubelka–Munk function, where F(R) = (1 − R)^2^/2R and R was the observed diffuse reflectance, the band gap value of ZnO was evaluated at approximately 3.1 ± 0.1 eV while the band gap of CuO was estimated at approximately 1.6 ± 0.2 eV.

**Figure 8:**
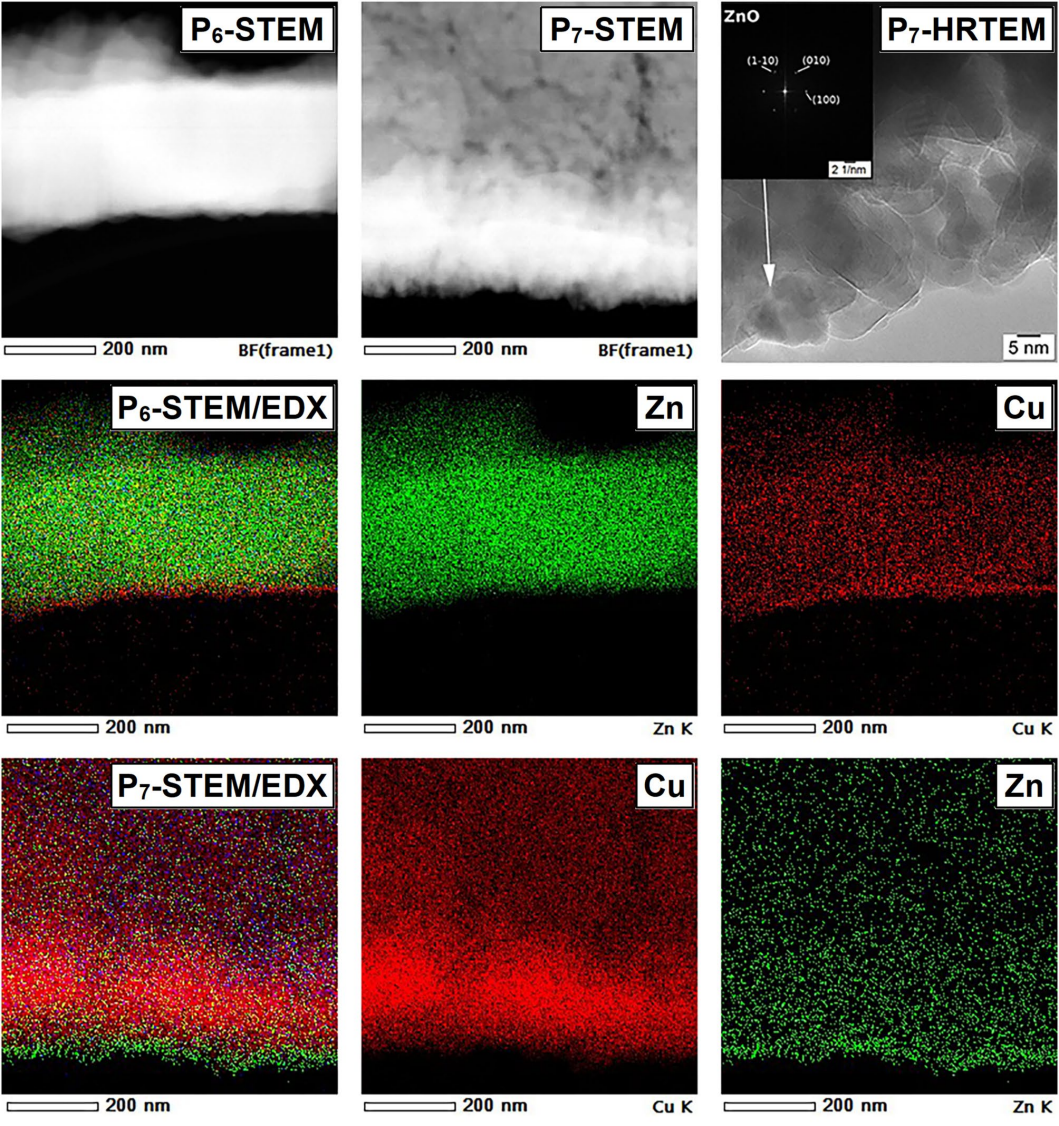
STEM images and EDX elemental mappings including the spatial distribution of the Zn and Cu elements in the functionalized ESMs (P_6_ and P_7_), HRTEM image and SAED pattern exhibiting the ZnO crystalline structure (inset) in P_7_ sample.

Further, in order to evidence the formation of the p–n junction between the ZnO and CuO, both functionalized ESMs containing a combination of the two metal oxides, meaning ZnO-coated ESM decorated with CuO nanoparticles (P_6_ sample) and CuO-coated ESM decorated with ZnO nanoparticles (P_7_ sample) were investigated by various TEM techniques, the obtained data being shown in Fig. 8. The samples for the TEM measurements were prepared by crushing the functionalized ESM in a mortar and dispersed in ethanol, a droplet of this suspension being deposited on TEM grids. Thus, the STEM images and the EDX elemental mappings of P_6_ and P_7_ samples put in evidence the formation of the CuO-ZnO p–n junction by the presence of Cu K and Zn K elements. It can be observed the spatial distribution of these two elements in the p–n junction: in P_6_ sample, Zn K is presented in the inner part and Cu K in the outer part while in P_7_ sample, Cu K is presented in the inner part and Zn K in the outer part. These results confirm the formation of p–n junction in two configurations in accordance with the metal oxide deposition sequences involved in the preparation of P_6_ and P_7_ samples. Moreover, the thickness of the outer layer (Cu K for P_6_ and Zn K for P_7_) was estimated at ~ 20–30 nm, in agreement with the cross-section FESEM images (Fig. 2). The HRTEM image acquired on P_7_ sample revealed that the CuO-ZnO p–n junction is formed by ZnO and CuO crystallites, the corresponding SAED pattern exhibiting the crystalline hexagonal wurtzite structure of the ZnO outer layer in agreement to the XRD data (Fig. 5).

The biocompatibility of the native and functionalized ESMs was evaluated in relation to human mesenchymal AFS cells by applying standard protocols for fluorescence microscopy, MTT assay and GSH assay.

**Figure 9:**
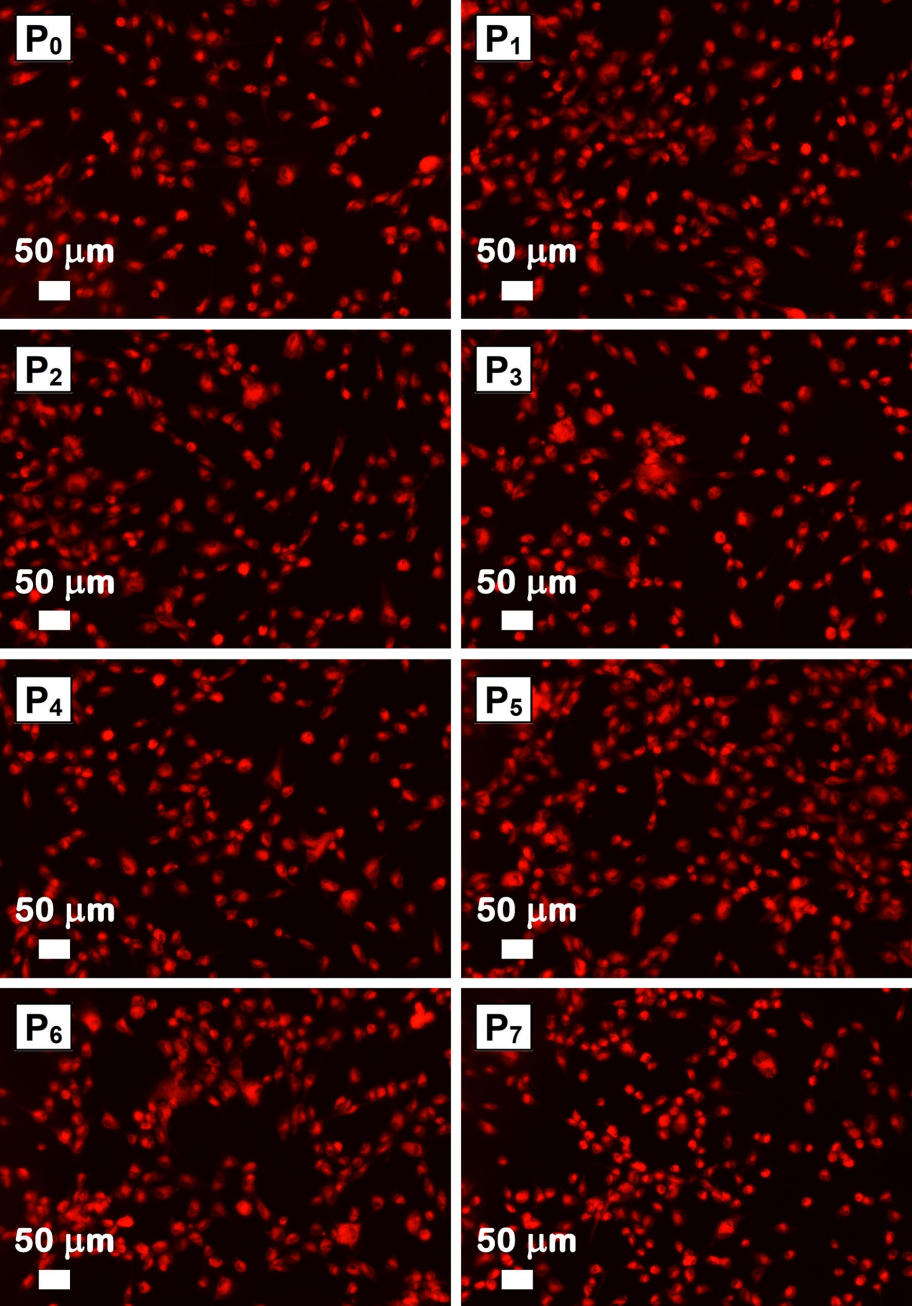
Fluorescence microscopy images of cells cultured on the native ESM (P_0_) and functionalized ESMs (P_1_–P_7_).

The fluorescent images from Fig. 9 evidence that the mesenchymal AFS cells cultured on the investigated samples have similar morphological features with the control sample (Figure S4). After 5 days in the presence of the functionalized ESMs, the cells incorporating the fluorescent CMTPX dye into the cytoplasm prove that the cells are viable, with an active metabolism. Also, the fluorescent images of the analyzed area reveal the adhesion and homogeneous distribution of the mesenchymal AFS cells on the surface of the investigated samples.

**Figure 10:**
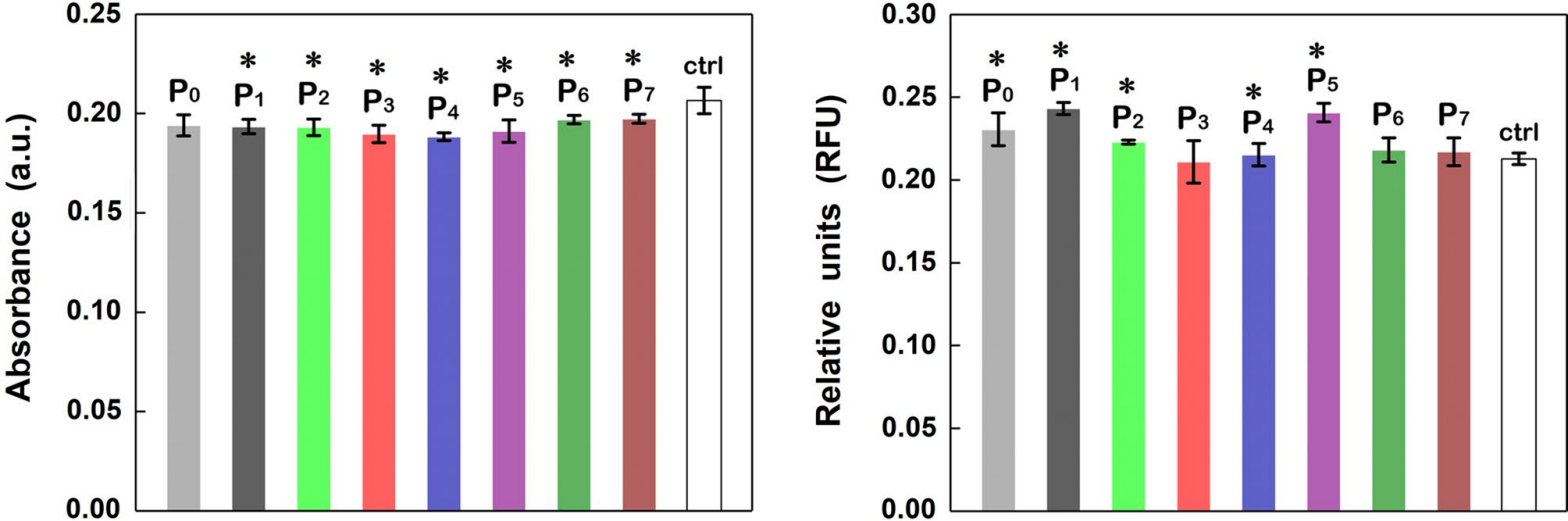
MTT (left) and GSH (right) analysis of the native ESM (P_0_) and functionalized ESMs (P_1_–P_7_). Statistical significance was assessed using two-tailed Studentʼs t-test; the results are represented as mean ± standard error, *P < 0.05 compared to control, n = 3.

Further, the cell viability and proliferation of the investigated samples were tracked by the MTT assay and the oxidative stress caused by these materials by GSH assay, the results being displayed in Fig. 10 left and Fig. 10 right, respectively. In the MTT assay, the recorded intensity for absorbance at 570 nm is proportional to the enzymatic activity of the cells consequently with their viability. In this context, the MTT tests confirmed that after 72 h of incubation, the mesenchymal AFS cells have a normal metabolism and growth in the presence of all functionalized ESM since only a smaller decrease, within the limit of 9% of the absorbance values, is noted in comparison with that related to the control sample. In the GSH assay, the amount of a natural antioxidant, glutathione, produced by cells during a normal functioning process was estimated, a high value recorded for this parameter indicating pathological changes in the cells. In our case, the GSH tests indicate that after 24 h of incubation, the presence of any functionalized ESM does not induces significant oxidative stress in the mesenchymal AFS cells, the GSH level being slightly higher within the limit of 14% as compared with that associated to the control sample. In both cases, MTT and GSH assays, the low difference between the recorded data and those obtained for the control sample are not of significant biological value. Consequently, the functionalized ESMs are well accepted by the mesenchymal AFS cells with no significant cytotoxic effect, the result emphasizing the potential of using them as biocompatible materials.

**Figure 11:**
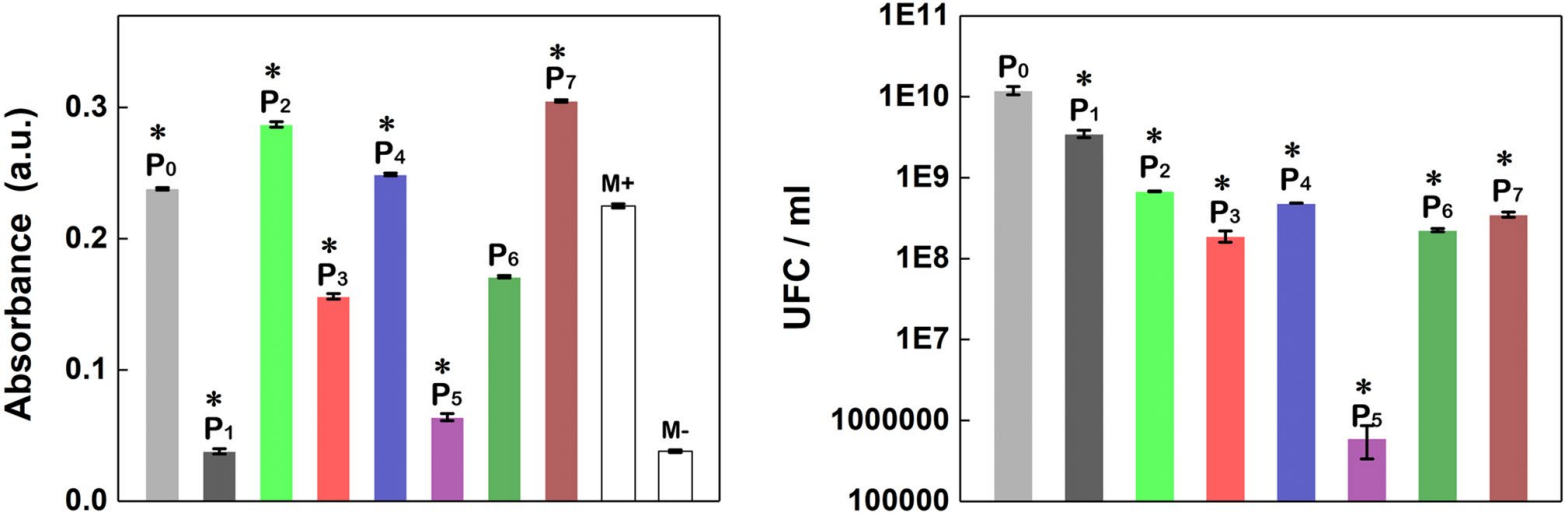
*E. coli* planktonic cells (left) and *E. coli* biofilm (right) inhibition growth analysis of the native ESM (P_0_) and functionalized ESMs (P_1_–P_7_). Statistical significance was assessed using two-tailed Studentʼs t-test; the results are represented as mean ± standard error, *P < 0.05 compared to control M + /M− = bacterial suspension/bacterial growth media, n = 3.

The antibacterial activity of native and functionalized ESM was investigated against *E. coli* in planktonic and biofilm states, the recorded values being presented in Fig. 11 left and Fig. 11 right, respectively.

*E. coli* was used in both states for comparing the interaction of the samples either with solitary planktonic cells or with biofilm bacteria, the latter being in fact an irregularly structured community of cells with a surface encased in an extracellular polymeric matrix^69^. Based on the data provided by both figures and without taking into account the native ESM, the following series regarding the antibacterial response of the functionalized ESMs are obtained: P_1_ > P_5_ > P_3_ > P_6_ > P_4_ > P_2_ > P_7_ in the case of *E. coli* planktonic state and P_5_ > P_3_ > P_6_ > P_7_ > P_4_ > P_2_ > P_1_ in the case of *E. coli* biofilm state. It has to be emphasized the particular behavior of Ag-coated ESM (P_1_), meaning that *E. coli* biofilms are more resistant to silver inhibition than *E. coli* planktonic cells. The result is consistent with previous data reported on the interactions of nanosilver with *E. coli* ^70^. Nevertheless, CuO-coated ESM decorated with Ag nanoparticles (P_5_) and CuO-coated ESM (P_3_) are the two samples that present a good antibacterial activity in the case of both *E. coli* states. The antibacterial response of these samples can be assessed based on the charge carriers produced by this semiconductor.

In order to improve the antibacterial activity of the ESM functionalized with metal oxides (P_2_, P_3_, P_6_ and P_7_ samples) through the activation of a p–n junction under illumination, these samples were exposed 3, 6 and 9 h to the visible light given by a commercially LED lamp, the result being shown in Table 1. Hence, on 9 h exposure, the reduction in bacterial growth increases from ~ 40% to ~ 54% for ZnO-coated ESM (P_2_) and from ~ 33% to ~ 52% for CuO-coated ESM (P_3_). Meanwhile, on 9 h exposure, the reduction in bacterial growth increases from ~ 5% to ~ 45% for ZnO-coated ESM decorated with CuO nanoparticles (P_6_) and from ~ 27% to ~ 82% for CuO-coated ESM decorated with ZnO nanoparticles (P_7_).

**Table 1:** Antibacterial efficiency of functionalized ESMs (P_2_, P_3_, P_6_ and P_7_) against *E. coli* under exposure to visible light.

Sample exposed to visible light	3 h			6 h			9 h		
	CFU ml^−1^	LR	R %	CFU ml^−1^	LR	R %	CFU ml^−1^	LR	R%
P_2_	2 × 10^5^	0.224	40.24	1 × 10^5^	0.279	47.37	4 × 10^5^	0.336	53.85
P_3_	2 × 10^5^	0.173	32.93	1 × 10^5^	0.200	36.84	5 × 10^5^	0.323	52.45
P_6_	3 × 10^5^	0.022	4.878	0.9 × 10^5^	0.309	50.88	5 × 10^5^	0.258	44.76
P_7_	2 × 10^5^	0.136	26.83	0.5 × 10^5^	0.525	70.18	2 × 10^5^	0.740	81.82

**Figure 12:**
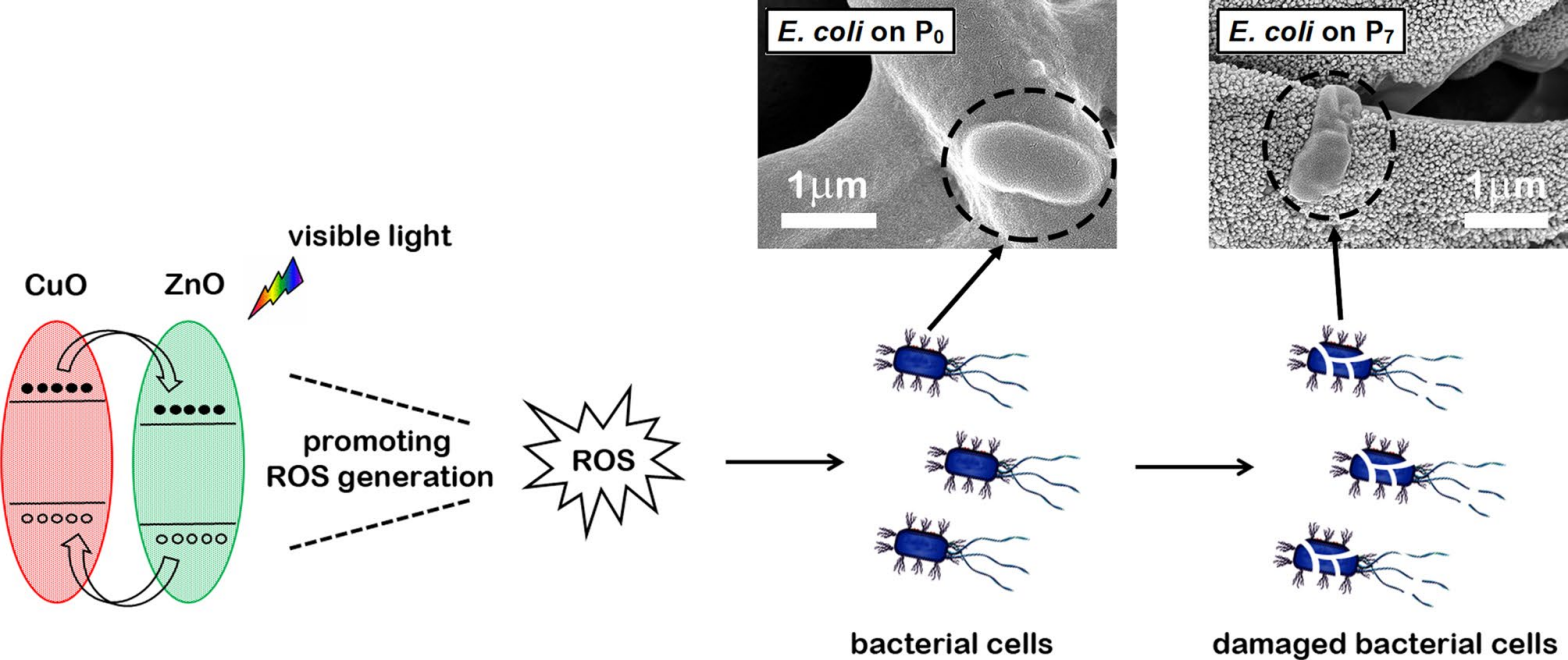
Schematic representation of the enhancement of antibacterial performance under visible light in the CuO-ZnO p–n junction. The FESEM images of an *E. coli* on the native ESM (P_0_) and on the functionalized ESM (P_7_).

According with the schematically representation from Fig. 12, the strongest bactericide effect observed in the last mentioned sample can be explained based on its particular architecture consisting in a compact inner absorbing layer (CuO, a semiconductor with a narrower bandgap) and a non-continuous outer window layer formed by nanoparticles (ZnO, a semiconductor with a wider band gap). The configuration of this axial p–n junction favours a high absorption of the visible light in the uncovered regions on the CuO compact layer and an efficient charge separation process induced by type II band alignment (staggered gap) formed at the interface between the two metal oxides^40,71^. Further, the generated charge carriers in the semiconductor materials can promotes the formation on their surface of ROS species, these being toxic to bacteria (by various mechanisms leading to the cell membrane rupture)^72,73^.

An important aspect regarding the stability of the CuO-ZnO p–n junction must be emphasized: no significant photo-corrosion effect was observed after the illumination with the LED lamp. The FESEM images and EDX data (Figure S5) obtained on the CuO-coated ESM decorated with ZnO nanoparticles (P_7_ sample) after visible light irradiation shown that the inorganic nanoparticles kept their shape and the fact that the Zn is presented almost in the same atomic percentage with that evaluated in the same sample before illumination.

Additionally, the change induces in the *E. coli* morphology by the antibacterial activity of the p–n junction under visible light can be observed in the FESEM images of this bacteria on the native ESM (P_0_ sample) and on the CuO-coated ESM decorated with ZnO nanoparticles (P_7_ sample). Thus, on P_0_ sample, the healthy *E. coli* is featured by its characteristic shape, a rod with spherical caps, with a smooth and intact surface, while on P_7_ sample, the dead *E. coli* revealed an irregular shape due to the membrane collapse.

The outcome of the experiments confirms that an efficient bactericide effect can be achieved, under visible light irradiation, in the case of the ESM functionalized with low cost and abundant metal oxides (ZnO, CuO) by employing a cost effective and scalable deposition technique (RF magnetron sputtering).

## Conclusions

Eggshell membranes (ESMs) were functionalized with inorganic materials (individual or a combination of Ag, ZnO, CuO) by RF magnetron sputtering, a straightforward deposition technique. The ESM was first coated with a Ag, ZnO or CuO continuous layer, further the metal oxide-coated ESMs being decorated with Ag nanoparticles while other ZnO-coated ESM and CuO-coated ESM being decorated with CuO and ZnO nanoparticles, respectively. The typical 3D porous network of the ESM is successfully preserved during its functionalization, the surface of the organic fibers being coated with a continuous and uniform inorganic film. Physico-chemical characterization of the functionalized ESMs confirms the presence of the inorganic components (Ag, ZnO and CuO) on the ESM surface. The cytotoxicity of functionalized ESMs was evaluated by fluorescence microscopy, MTT assay and GSH assay, the recorded data evidencing their biocompatibility potential, the materials being well accepted by the mesenchymal AFS cells with no significant cytotoxic effect. Further, the antibacterial activity of functionalized ESMs was analyzed against *E. coli* in planktonic and biofilm states. A particular behavior was observed in the case of Ag-coated ESM (P_1_), *E. coli* biofilms being more resistant to silver inhibition than *E. coli* planktonic cells. From all functionalized ESMs, CuO-coated ESM decorated with Ag nanoparticles (P_5_) and CuO-coated ESM (P_3_) are the two samples that present a good antibacterial activity in the case of both *E. coli* states. Under exposure to visible light from a commercially LED lamp, the CuO-coated ESM decorated with ZnO nanoparticles (P_7_) exhibits improved antibacterial performance due to the formation of an axial p–n junction between the two metal oxides which enhances the generation of highly reactive oxygen species favoring the bacteria killing. The outcome of this study proves that by tailoring specific properties, materials with enhanced antibacterial properties under visible light can be fabricated. Using a cost-effective and environmentally safe path which involves an abundant industrial bio-waste (ESM), low-cost metal oxides (ZnO and CuO) and a straightforward deposition technique (RF magnetron sputtering) is an added benefit. Such materials can be excellently suited as a component for affordable wound dressing with intense antibacterial activity.

## Supplementary information

Supplementary Information

## Data Availability

The datasets supporting the conclusions of the current study are presented in the manuscript and supporting information.
