# Supplementary material for: Functionalization of eggshell membranes with CuO–ZnO based p–n junctions for visible light induced antibacterial activity against Escherichia coli
Source: Sci Rep. 2020 Dec 1;10:20960. doi: 10.1038/s41598-020-78005-x (PMC7708484; doi:10.1038/s41598-020-78005-x)
Supplement: Supplementary file 1 — Supplementary Information [file 41598_2020_78005_MOESM1_ESM.docx]

Functionalization of eggshell membranes with CuO-ZnO based p-n junctions for visible light induced antibacterial activity against Escherichia coli

Nicoleta Preda^1¥*^, Andreea Costas^1¥^, Mihaela Beregoi^1¥^, Nicoleta Apostol^1^, Andrei Kuncser^1^, Carmen Curutiu^2^, Florin Iordache^3^, Ionut Enculescu^1**^

^1^National Institute of Materials Physics, Atomistilor 405A, 077125, Magurele, Romania

^2^University of Bucharest, Faculty of Biology, Microbiology Immunology Department, Aleea Portocalelor 1-3, 060101, Bucharest, Romania

^3^University of Agronomic Sciences and Veterinary Medicine of Bucharest, 011464 Bucharest, Romania.

^¥^ Nicoleta Preda, Andreea Costas and Mihaela Beregoi contributed equally to this work.

^*^Corresponding author: Nicoleta Preda

Tel (office): + 40 21 3690185

Fax (office): + 40 21 3690177

e-mail: [nicol@infim.ro](mailto:nicol@infim.ro)

^**^Corresponding author: Ionut Enculescu

Tel (office): + 40 21 3690185

Fax (office): + 40 21 3690177

e-mail: [encu@infim.ro](mailto:encu@infim.ro)

| Sample | Element, at. % | | | | | | |
| --- | --- | --- | --- | --- | --- | --- | --- |
|  | C | N | O | S | Ag | Zn | Cu |
| P_1_ | 12.23 | 12.69 | 55.23 | 2.15 | 17.7 | - | - |
| P_2_ | 7.04 | 6.08 | 47.01 | 3.6 | - | 36.27 | - |
| P_3_ | 7.64 | 7.04 | 44.36 | 3.34 | - | - | 37.62 |
| P_4_ | 6.54 | 7.01 | 49.77 | 3.34 | 0.95 | 32.39 | - |
| P_5_ | 7.83 | 7.77 | 44.59 | 3.69 | 1.36 | - | 34.76 |
| P_6_ | 7.88 | 7.67 | 40.81 | 4.16 | - | 38.2 | 1.28 |
| P_7_ | 7.13 | 7.95 | 47.63 | 2.89 | - | 0.89 | 33.51 |

Table S1. EDX elemental analysis (atomic percentage) of the

functionalized ESMs (P_1_-P_7_).


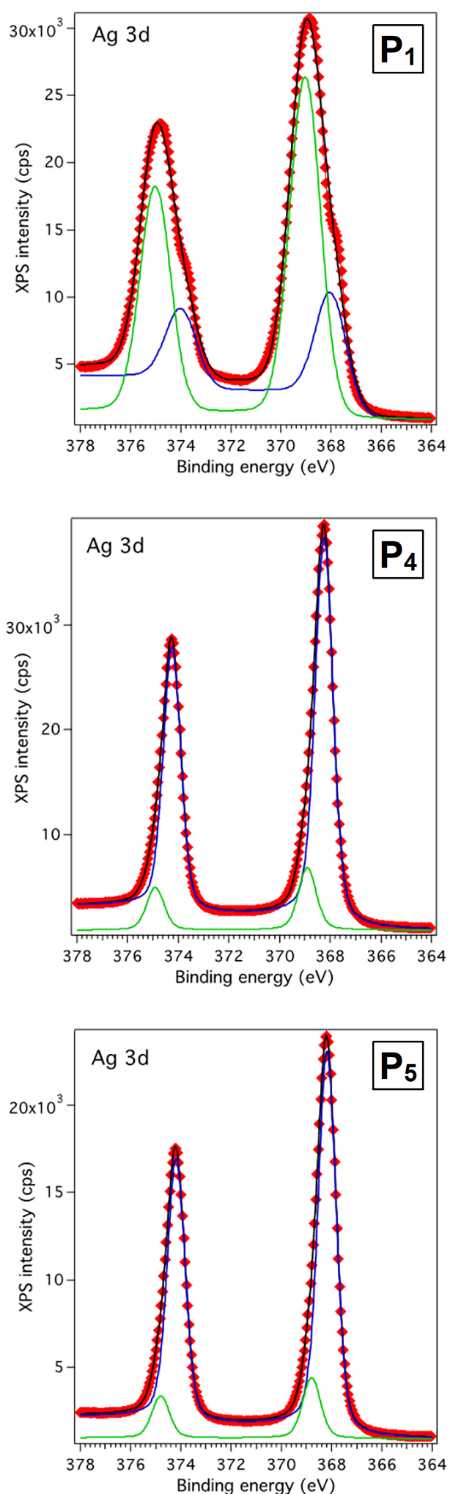


Figure S1. High resolution XPS spectra of the core level Ag 3d for

functionalized ESM (P_1_, P_4_ and P_5_).


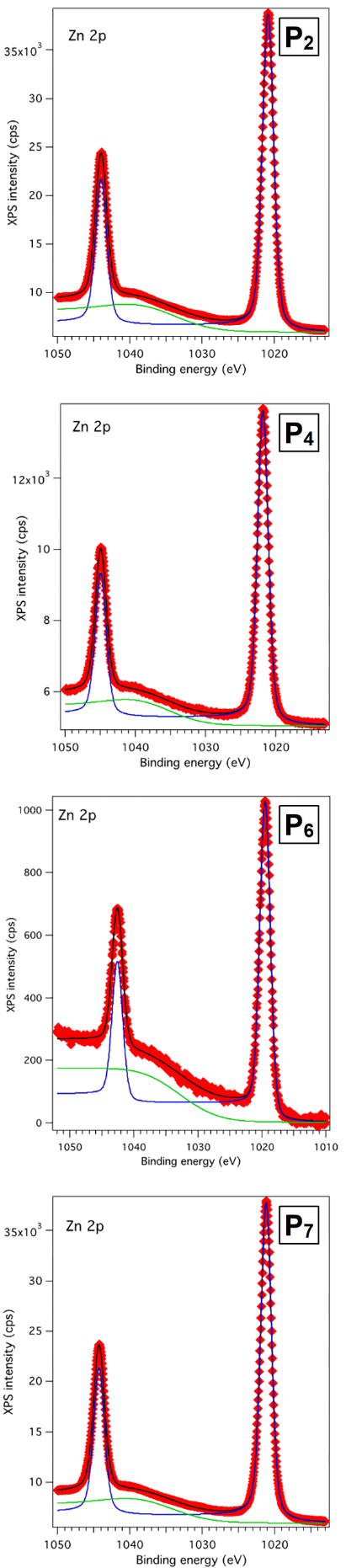


Figure S2. High resolution XPS spectra of the core level Zn 2p for

functionalized ESM (P_2_, P_4_, P_6_ and P_7_).


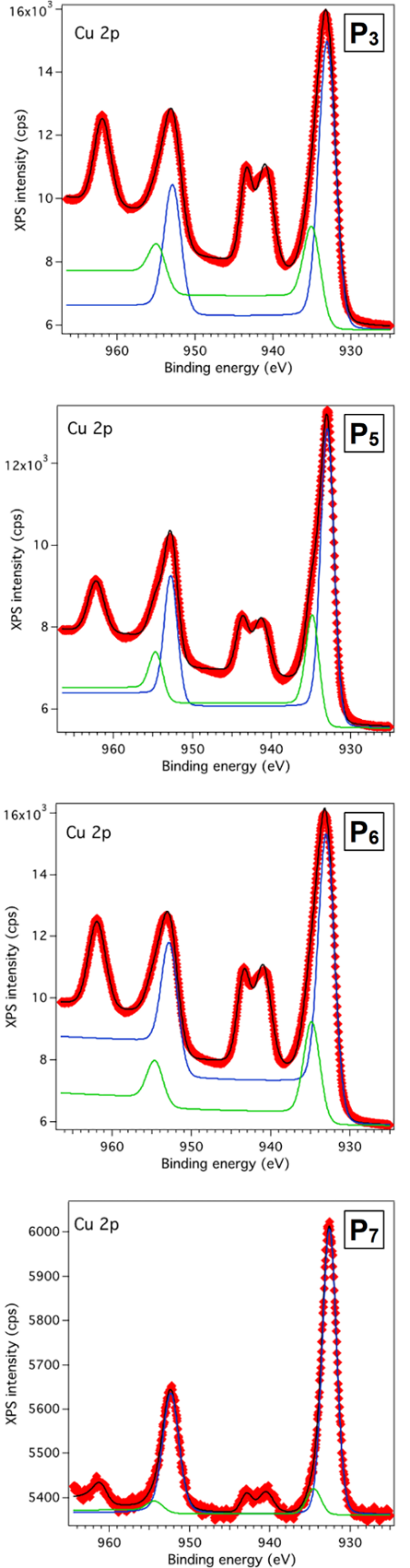


Figure S3. High resolution XPS spectra of the core level Cu 2p for

functionalized ESM (P_3_, P_5_, P_6_ and P_7_).


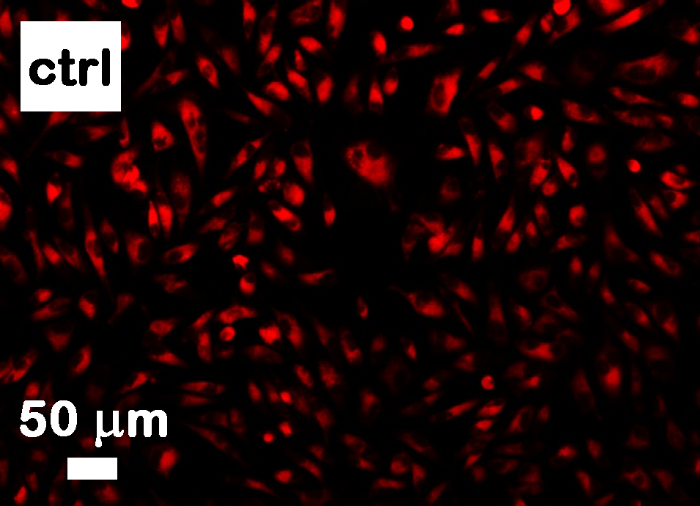


Figure S4. Fluorescence microscopy images of control sample.


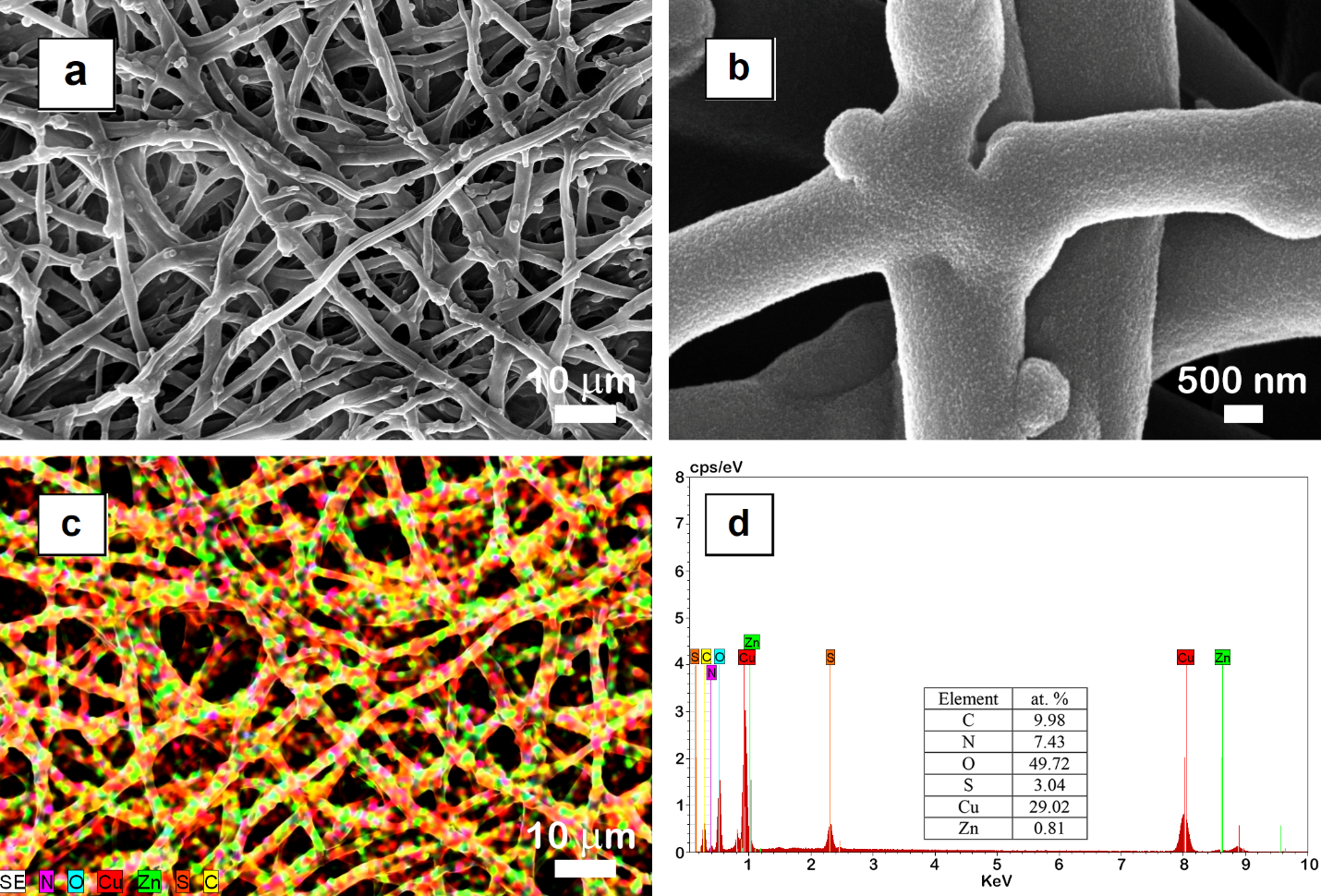


Figure S5. FESEM images at two magnifications (a, b), EDX mapping (c) and corresponding EDX spectrum (d) of the functionalized ESM (P_7_) after 9 h illumination with visible light.
